# Novel Microdilution Method to Assess Double and Triple Antibiotic Combination Therapy* In Vitro*

**DOI:** 10.1155/2016/4612021

**Published:** 2016-04-18

**Authors:** Mohamed El-Azizi

**Affiliations:** Faculty of Pharmacy and Biotechnology, Department of Microbiology, Immunology and Biotechnology, German University in Cairo, New Cairo City, Cairo 11835, Egypt

## Abstract

An* in vitro* microdilution method was developed to assess double and triple combinations of antibiotics. Five antibiotics including ciprofloxacin, amikacin, ceftazidime, piperacillin, and imipenem were tested against 10 clinical isolates of* Pseudomonas aeruginosa*. Each isolate was tested against ten double and nine triple combinations of the antibiotics. A 96-well plate was used to test three antibiotics, each one alone and in double and triple combinations against each isolate. The minimum bacteriostatic and bactericidal concentrations in combination were determined with respect to the most potent antibiotic. An Interaction Code (IC) was generated for each combination, where a numerical value was designated based on the 2-fold increase or decrease in the MICs with respect to the most potent antibiotic. The results of the combinations were verified by time-kill assay at constant concentrations of the antibiotics and in a chemostat. Only 13% of the double combinations were synergistic, whereas 5% showed antagonism. Forty-three percent of the triple combinations were synergistic with no antagonism observed, and 100% synergism was observed in combination of ciprofloxacin, amikacin, and ceftazidime. The presented protocol is simple and fast and can help the clinicians in the early selection of the effective antibiotic therapy for treatment of severe infections.

## 1. Introduction

Empirical antibiotic combination therapy is employed to treat severe infections in neutropenic and severely ill patients when the antibiotic susceptibility profile of the causative pathogen is unknown. Combined antibiotic therapy is preferred over monotherapy because it has a broader antibacterial spectrum, synergistic effect, and reduced risk of emerging resistance during therapy [1]. It is recommended to narrow treatment to the most efficient antibiotic once the identification and susceptibility of the pathogen are known, consequently limiting the duration of therapy [2–4]. The length of therapy could also be shortened when using appropriate drug combination with synergistic effect which results in rapid killing of the pathogen [1, 5, 6].

In patients with severe sepsis, the decision of selection of the combination therapy must be taken in the first hours of diagnosis [7]. It has been reported that the initial use of proper combination therapy improves outcomes in patients with sepsis or ventilator-associated pneumonia (VAP) caused by Gram-negative bacteria [3, 8–10].

Combined antibiotic therapy is commonly used against multidrug-resistant (MDR) Gram-negative organisms, which have emerged as a major threat to hospitalized patients with mortality rates ranging from 30 to 70% [3]. Infections associated with MDR* Pseudomonas aeruginosa*,* Acinetobacter baumannii*, and Enterobacteriaceae have a substantial impact on hospital costs and mortality rates [11].

Optimization of the therapy for* Pseudomonas* spp. is challenging because of the ability of the bacteria to develop multiple mechanisms of resistance [3, 12, 13]. The successful treatment of infections caused by* P. aeruginosa* remains poor with a crude mortality rate of as high as 50% [3, 14, 15].

*In vitro* drug combination is routinely assessed by checkerboard assay, which is suitable for dual antibiotic therapy. Time-kill assay is used to evaluate the bactericidal activity of two or more antibiotics when used against a particular pathogen. An abbreviated three-dimensional checkerboard assay, which is complicated and time-consuming, was developed to test triple antibiotic combinations [16]. The E-test is also useful tool for testing antimicrobial combinations that can provide clinicians with proper treatment options [17].

This study aims at developing an* in vitro* rapid and simple method to assess double and triple antibiotic combination therapy. This model will be helpful in the early selection of the best antibiotic combinations and accordingly limits the duration of the treatment. To test the model, five antibiotics including ciprofloxacin, amikacin, ceftazidime, piperacillin, and imipenem were selected. The antibiotics were tested in double and triple combinations against 10 clinical isolates of* P. aeruginosa*; some of them are MDR.

## 2. Materials and Methods

### 2.1. Chemicals and Reagents

Unless otherwise indicated, all chemicals and reagents were purchased from Sigma-Aldrich Chemical Co., Saint Louis, Missouri, USA.

### 2.2. Antibiotics

Amikacin (AMK) and piperacillin (PIP) were purchased from Sigma-Aldrich Chemical Co., Saint Louis, Missouri, USA. Ciprofloxacin (CIP) was provided by Bayer Corporation, Germany. Ceftazidime (CAZ) was provided by GlaxoSmithKline, NC, USA. Imipenem (IMP) was provided by Merck Research Laboratories, NJ, USA.

### 2.3. Microorganisms

Ten clinical isolates of* P. aeruginosa* were used in the study. The isolates were identified to species level using the standard microbiological techniques.

### 2.4. Antimicrobial Susceptibility

The minimum inhibitory concentrations (MICs) of the tested antibiotics were determined by broth microdilution method as described in the Clinical and Laboratory Standards Institute (CLSI) guideline [18]. The minimum bactericidal concentrations (MBCs) were determined by mixing the contents of each well at MIC and higher concentrations, and 10 *μ*L portions were then taken from each well and streaked onto the surface of blood agar. After 24 h incubation, the number of colony forming units per milliliter (CFU/mL) was counted and the MBCs that kill 99.9% of bacteria were determined.

### 2.5. Evaluation of Double and Triple Antibiotic Combinations* In Vitro*

Each isolate was tested against ten double and nine triple combinations of the antibiotics. Three groups of double combination were tested including CIP with AMK or *β*-lactam, AMK with each of the *β*-lactams, and the *β*-lactam antibiotics with each other. For triple therapy, all possible combinations of CIP with other antibiotics were tested.

One 96-well plate (BD Falcon, USA) was used to test three antibiotics at a time, each one alone and in double and triple combinations against single isolate of* P. aeruginosa* (Table 1). Briefly, rows A to C were used for each of the antibiotics alone. Rows D to F were used to test double combinations of the first and second, first and third, and second and third antibiotics, respectively. Row G was used for the triple combination of the antibiotics. All wells were filled with 50 *μ*L of cation-adjusted Muller Hinton broth (MHB). Fifty microliters of the antibiotics at 4x of the highest tested concentrations in MHB (alone and in double or in triple combinations) was delivered to wells A11–G11. Twofold serial dilutions were made by using multichannel pipettes from wells A11–G11 to A1–G1 and 50 *μ*L portions were discarded from the last column. Bacterial suspensions (50 *μ*L) were added to all wells to bring the total volume to 100 *μ*L and initial inoculum size of 1 to 5 × 10^5^ CFU/mL. Antibiotic-free wells in column A12–G12 were used as positive control. Plates were placed on plate shaker for 30 minutes followed by incubation at 37°C for 24 h. The minimum bacteriostatic and bactericidal concentrations in combinations were determined with respect to the most potent antibiotic. The experiments for each isolate were carried out in triplicate, and the results were considered only in case of agreement of the MIC in at least two out of three wells.

### 2.6. Assessing the Antibiotic Combinations

The combination was assessed with respect to the most potent antibiotic, with lowest MIC value, alone and in double and triple combinations with other antibiotics. An Interaction Code (IC) was generated for each combination, where a numerical value was designated based on the 2-fold increase or decrease in the MICs of the most potent antibiotic in combination. In double combination, the interaction type (IT) is defined as synergistic (S) if the IC value is 2 or more, which indicates that the MIC of the most potent antibiotics decreased by 2-fold or more compared to the MIC of the most potent antibiotic alone. The interaction is indifferent (I) if the IC value is zero, −1, or 1 which indicates that the MIC of the most potent antibiotic was unchanged or increased or decreased by one-fold concentration, respectively, in the combinations. The interaction is antagonistic (A) if the IC value is −2 or less which indicates that the MIC of the potent antibiotic increased by 2-fold or more in combination with other antibiotics. In triple combination, the same rule is applied, where the interaction is considered synergistic if the IC value is 2 or more and higher than the IC values of any of the double combinations which contain the potent antibiotic. The interaction is defined as antagonistic if the IC value is −2 or less and lower than the IC values of any of the double combinations which contain the most potent antibiotic. Triple combination is defined as indifferent if the IC value is zero, −1, or 1 or equal to the IC values of the double combinations which contain the most potent antibiotic.

The results of double combination obtained by the presented technique were verified by the checkerboard method. The combination response was evaluated by calculation of the friction inhibitory index (FIC) as follows:

(1) FIC indexFICA+FICB=MIC of drug A,in combinationMIC of drug A,tested alone+MIC of drug B,in combinationMIC of drug B,tested alone.

The interaction is defined as synergistic if the FIC index is 0.5 or less, indifferent if the FIC index is more than 0.5 and less than 4, and antagonistic if the FIC index is more than 4 [19].

### 2.7. Evaluation of the Double and Triple Antibiotic Combinations by Time-Kill Assay* In Vitro*

To verify the results obtained by the microdilution method, the bactericidal activity of CIP, AMK, and CAZ alone and in double and triple combinations was determined by time-kill assay. Briefly, 24-hour-old culture of isolate PA14 was suspended in normal saline and used to inoculate 100 mL of cations-adjusted MHB in 250 mL Erlenmeyer flasks to give initial inoculum size of 1 to 5 × 10^5^ CFU/mL. The antibiotics were added alone and in double or triple combinations at concentrations equivalent to 1/4 or 1/2 of their MICs. The flasks were incubated at 37°C and 100 rpm. Samples were taken at different time intervals to measure the viability of the bacteria calorimetrically using XTT [3-(4,5-Dimethylthiazol-2-yl)-2,5-Diphenyltetrazolium Bromide] as described before [20]. In brief, 1 mL samples were removed and centrifuged at 10,000 rpm for 10 minutes. One milliliter aliquots of XTT with menadione were added to the resultant pellets to obtain final concentrations of 1.0 mg of XTT/mL and 50 *μ*M menadione. The samples were incubated in the dark for 1 h at 37°C, after which a colorimetric change in the XTT was measured using a microtiter plate reader at 490 nm. Drug-free experiments were used as control.

### 2.8. Evaluation of Double and Triple Antibiotic Combinations by Time-Kill Assay in a Chemostat

**Figure 1:**
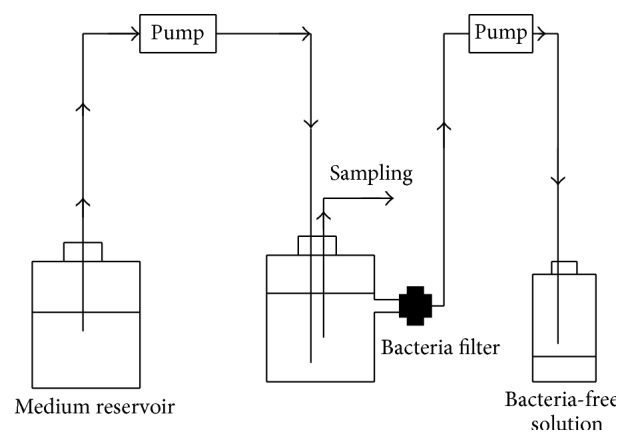
Configuration of the chemostat-like model to evaluate double and triple antibiotic combinations by time-kill assay* in vitro*. The bottles were connected to two IV infusion pumps via IV infusion sets. The first pump delivers fresh medium to the bottles at 10 mL/h to provide nutrients and continuously dilute the antibiotics. A second pump was connected to the bottle through disposable syringe bacterial filter to withdraw the medium without the bacteria at 10 mL/h flow rate.

The experiment was configured to simulate the* in vivo* situation in which bacteria are exposed to supra-MIC and sub-MIC of the antibiotics (Figure 1). The tested isolate PA14 was added to bottles containing cation-adjusted MHB to give initial inoculum size of 1 to 5 × 10^5^ CFU/mL. The first doses of the antibiotics, alone or in combinations, were added at concentrations equivalent to 2x of their MICs (peak concentration at time of delivery) to the bottles after 10 hours of inoculation. The bottles were connected to two IV infusion pumps via IV infusion sets. The first pump delivers fresh medium to the bottles at 10 mL/h to provide nutrients and continuously dilute the antibiotics. A second pump was connected to the bottle through disposable syringe bacterial filter (0.45 *μ*m pore size) to withdraw the medium without the bacteria at 10 mL/h. The bottles were placed over magnetic stirrer at 37°C. Second and third doses of the antibiotics were added at 30 and 60 h and the flow rate was kept at 10 mL/h throughout all experiments. To avoid clogging of the filter, 2 mL portions of the fresh medium were injected into the filter in opposite direction to remove any bacterial cells back to the bottles, and the filters were replaced when needed. At different time intervals, 1 mL samples were taken for determination of the viable cell by viable count. Drug-free experiments were used as control.

### 2.9. Statistics Analysis

Each experiment was performed in triplicate and the mean and the Standard Deviation (SD) were calculated. One-way analysis of variance (ANOVA) was used to determine the differences between various treatments. Tukey's pair comparison test was used at the chosen level of probability (*p* < 0.05) to determine significant difference between means.

## 3. Results

### 3.1. Antimicrobial Susceptibility

The tested isolates showed variable susceptibility to the antibiotics (Table 2). Six isolates were susceptible to all antibiotics, two isolates, PA11 and PA20, were resistant to CIP, CAZ, PIP, and IMP, and one isolate, PA14, was resistant to CIP and PIP. Amikacin was effective against all tested isolates with MIC range of 2–8 *μ*g/mL. Excluding the resistant isolates, CIP was the most potent antibiotic, with MIC range of 0.03–0.5 *μ*g/mL. The MIC range for CAZ, PIP, and IMP against the susceptible isolates was 1–8, 8–16, and 2–8 *μ*g/mL, respectively.

### 3.2. Evaluation of Double and Triple Combinations of the Antibiotics

A total of nineteen sets of antibiotic combinations, 10 double combinations and 9 triple combinations, were tested against the isolates of* P. aeruginosa*. With triple therapy, 43% of the combinations were synergistic with no antagonism observed in all interactions (Tables 3 and 4). Only 13% of the double combinations were synergistic, whereas 5% showed antagonism. Isolates PA11 and PA20 were resistant to 85% of the double combinations which contain one or two antibiotics to which the isolates were resistant, but they were susceptible to triple combinations which include one or both drugs to which the isolates were resistant. When amikacin was included in triple combination, the resistant isolates were susceptible to the antibiotics.

The best combination was obtained in triple combination of CIP, AMK, and CAZ, where synergism was observed with all isolates followed by 80 and 70% in combination of AMK with PIP and CAZ and with CIP and PIP, respectively (Table 5).

The checkerboard assay was used to verify the result of the double combinations obtained by the presented model. The FIC index was 0.5 or less (synergism) in 15 isolates (data not shown) compared to 13 isolates with the presented model, where the IC value was 2 or more in double combination of the antibiotics. Antagonism was demonstrated in addition of IMP to either CAZ or PIP in 5% of the combinations similar to the results obtained by the presented microdilution model.

### 3.3. Evaluation of Double and Triple Combinations of the Antibiotics by Time-Kill Assay

**Figure 2:**
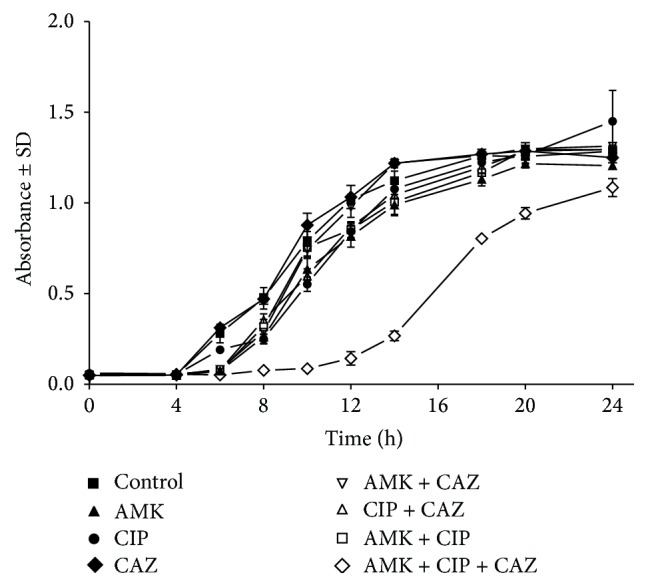
Evaluation of double and triple combinations of ciprofloxacin, amikacin, and ceftazidime against isolate PA14 by time-kill assay* in vitro*. Isolate PA14 was used to inoculate 100 mL of cations-adjusted Muller Hinton broth in 250 mL Erlenmeyer flasks to give initial inoculum size of 1 to 5 × 10^5^ CFU/mL. The antibiotics were added alone in double or triple combinations at concentrations equivalent to 1/2 of their MICs. AMK, amikacin; CIP, ciprofloxacin; CAZ, ceftazidime.

Isolate PA14 was selected to verify the* in vitro* antibiotic synergism using time-kill assay. The double combinations of the antibiotics (CIP, AMK, and CAZ) were found to be additive (IC = 1). Triple combination of the antibiotics, on the other hand, was synergistic (IC = 3). The time-kill assay showed that the double and triple combinations of the antibiotics at their 1/4 MICs had no significant effect (*p* > 0.5) on the bactericidal activity compared to each antibiotic alone (data not shown). When the drugs were tested at their 1/2 MICs, triple combination of the antibiotics significantly inhibited the growth of the bacteria (*p* < 0.05) compared to each drug alone and to all sets of double combination (Figure 2).

### 3.4. Evaluation of the Double and Triple Combinations of the Antibiotics by Time-Kill Assay in a Chemostat

**Figure 3:**
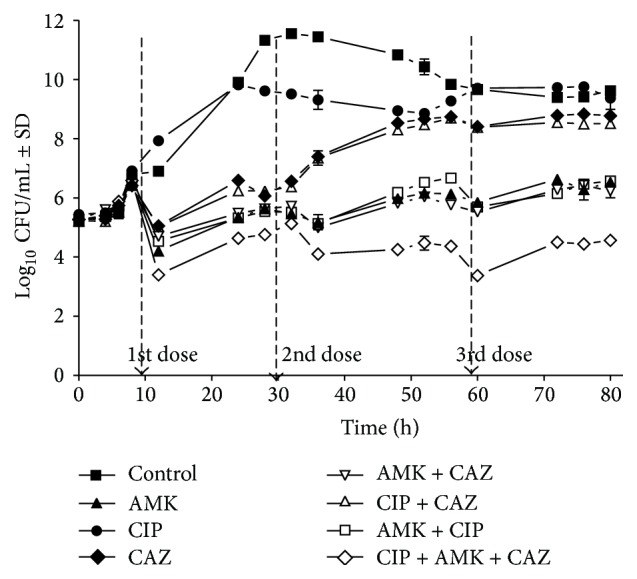
Evaluation of double and triple combinations of ciprofloxacin, amikacin, and ceftazidime against isolate PA14 by time-kill assay in a chemostat model. Isolate PA14 was added at 1 to 5 × 10^5^ CFU/mL to bottles containing cation-adjusted Muller Hinton broth. The first doses of the antibiotics, alone or in combination, were added at concentration equivalent to double of their minimum inhibitory concentrations after 10 hours of inoculation. The second and third doses of the antibiotics were added at 30 and 60 hours of incubation. The flow rate was kept at 10 mL/h throughout the experiment. AMK, amikacin; CIP, ciprofloxacin; CAZ, ceftazidime.

The experiment was configured to simulate the* in vivo* situation in which bacteria are exposed to supra-MIC and sub-MIC of the antibiotics. Fresh medium was continuously pumped to the bottles. The doses of the antibiotics alone and in combination were added after 10, 30, and 60 hours following inoculation with the isolate. Synergism was defined as reduction by 2-log or more of the CFU/mL of the bacteria when two or three antibiotics were added compared to the most potent antibiotic alone. AMK was the most potent antibiotic against the isolate when used alone compared to CIP and CAZ. Synergism was only demonstrated following the addition of three doses of the triple therapy, while all double combinations were indifferent (Figure 3).

## 4. Discussion

The emergence of infections caused by MDR Gram-negative pathogens is challenging for clinicians. These infections are responsible for high mortality rates, and few useful antimicrobial options are available for their treatment. The use of inappropriate antibiotic therapy and consequently the delay in starting effective treatment are the primary cause of poor outcomes in severe infections [22]. High mortality rates have been reported among patients diagnosed with nosocomial infections and who received empiric treatment while the* in vitro* susceptibility profile of the causative pathogen was unavailable [3].

Testing for antibiotic combination becomes a potentially powerful tool to help the clinicians in the selection of the appropriate antibiotic therapy. It will be useful if there is a simple protocol that can be experimentally used to test antibiotic combination parallel to the determination of susceptibility profile of the pathogen. This test becomes more necessary with the emergence of infections caused by MDR bacteria which may need treatment with two or more antibiotics.

The presented model of testing antibiotic combination is based on the microdilution technique. The method was adapted to test the susceptibility profile of a pathogen concurrently with testing of two or three antibiotic combinations in the same plate. The method can be modified to test more antibiotics by adding more plates. Compared with the checkerboard and time-kill assays, the presented protocol is simple and fast and needs fewer materials to test the combination of two or more antibiotics. Unlike the checkerboard assay, where one set of double combination is assessed in the whole plate, fewer combined concentrations, in one row of 11 wells, were used in the presented model with few proportions or ratios. Results obtained with different ratios may be significantly different [23]. One more advantage of the presented protocol is its ability to provide information about inhibitory and bactericidal effects of the antibiotic combination, which is necessary for comparing the results to the time-kill assay.

*P. aeruginosa* was selected as a model of Gram-negative bacteria to evaluate the presented method in assessing double and triple antibiotic combinations. The microorganism has constitutive resistance to most classes of antibiotics and can acquire resistance to all available treatment therapy. Treatment of* Pseudomonas* infection is mostly empirical until the culture is recovered, and susceptibility profile is obtained. High resistance rates and the observed high mortality would account for the use of inappropriate initial therapy [19]. Five antipseudomonal antibiotics were selected including ciprofloxacin, amikacin, and three *β*-lactam antibiotics (piperacillin, ceftazidime, and imipenem). The *β*-lactam antibiotics represent the penicillin, cephalosporin, and carbapenem antibiotic classes, respectively.

Two of the tested isolates were resistant to four antibiotics, and one isolate was resistant to two antibiotics (Table 2). Because empirical drug regimen may include antibiotics to which the pathogen may be resistant, it is beneficial to include isolates that are resistant to one or more antibiotics in the tested drug combinations.

Synergism was more common with triple combinations than with double combinations with no antagonism observed in all isolates compared to double combinations (Tables 3 and 4). This finding is in agreement with other studies that compared double and triple antibiotic combinations against* P. aeruginosa* using different assessment methods [23, 24].

In triple combination, the best outcome was obtained when AMK was included in the therapy. Synergism was found in all isolates when AMK was combined with CIP and CAZ, whereas 80 and 70% of the interaction were synergistic when the antibiotic was combined with PIP and CAZ and with CIP and PIP, respectively. Aminoglycosides induce outer membrane changes in* P. aeruginosa* and consequently increase uptake of other antibiotics in the combined therapy [25]. The results of the double combination generated by our model are in agreement with other previously published* in vitro* studies and are different from others [26–29]. This may be attributed to the differences in methods, antibiotic concentrations, bacterial inocula, and strain-dependent factors [1]. The results of double combination generated by our model and the checkerboard assay were comparable. Synergism and antagonism were demonstrated in 13 and 5% of the combinations, respectively, using the presented model compared to 15 and 5% using the checkerboard assay.

Synergism was demonstrated in 40% of the combination of CAZ with PIP, while antagonism was obtained in 30 and 20% of the combination of IMP with CAZ or PIP, respectively. Combinations of two *β*-lactam antibiotics may be beneficial in providing a synergistic activity or a broad spectrum of antibiotic coverage against specific pathogen in certain clinical cases [30]. Double *β*-lactam antibiotic therapy was found to be effective in treatment of febrile granulocytopenic patients [18, 31]. Synergism, indifference, and antagonism were previously reported in double combination of *β*-lactam antibiotics against pathogenic bacteria including* Pseudomonas* [30–32]. Synergistic response may result when each of the *β*-lactams targets different penicillin-binding proteins [30]. Antagonism was reported on double *β*-lactam combinations which involve imipenem, a potent inducer of* ampCβ*-lactamase genes, when tested against* Pseudomonas* [33, 34].

Two sets of time-kill assay were used to verify the results obtained by the presented model using isolate PA14. In one set, CIP, AMK, and CAZ were added at 1/4 or 1/2 of their MICs. In the second set of experiment, the antibiotics, alone or in combination, were tested in a chemostat at double of their MICs. Three doses of the drugs were added after 10, 30, and 60 hours of incubation, where their concentrations decreased over the time course of the experiment. This configuration simulates the* in vivo* condition in which bacterial growth precedes the antibiotic therapy and microorganism is exposed to supra-MIC and sub-MIC of the antibiotics. Synergism was demonstrated in the triple combination of the antibiotics when they were tested at 1/2 of their MICs (Figure 2). Synergism was also evident in the chemostat especially after the addition of the second and the third doses of the antibiotics in triple combination (Figure 3). It is possible to correlate the results obtained by the presented microdilution method and the time-kill assay to demonstrate synergistic response. The presented microdilution method has both inhibitory and bactericidal end points which can be compared with the killing result obtained by the time-kill assay. One disadvantage of the checkerboard technique is the poor correlation of the inhibitory indicator, FIC index, to the bactericidal result of the time-kill assay [25].

## 5. Conclusion

The presented* in vitro* model provides an easy and simple technique for the evaluation of antibiotic interaction especially when more than two antibiotics are used. Unlike the checkerboard and the time-kill assay methods, the presented model needs fewer materials, and one microtiter plate is enough to test three antibiotics when tested alone or in double or triple combinations. The results obtained by the presented method were reproducible and comparable to the data obtained by checkerboard method. The method can be a useful tool to help the clinicians in the selection of the appropriate antibiotic therapy and consequently avoid the delay in starting effective treatment of severe infections. Further work needs to be performed on larger numbers of* Pseudomonas* and other problematic Gram-negative and Gram-positive bacteria with wider selection of the antibiotics to verify the results obtained by the presented model.

## Figures and Tables

**Table 1:** Schematic diagram showing the distribution of tested antibiotics in the wells.

	1	2	3	4	5	6	7	8	9	10	11	12	
A	Drug A	Drug A	Drug A	Drug A	Drug A	Drug A	Drug A	Drug A	Drug A	Drug A	Drug A	Positive control (culture medium + organism)	A
B	Drug B	Drug B	Drug B	Drug B	Drug B	Drug B	Drug B	Drug B	Drug B	Drug B	Drug B		B
C	Drug C	Drug C	Drug C	Drug C	Drug C	Drug C	Drug C	Drug C	Drug C	Drug C	Drug C		C
D	Drugs A + B	Drugs A + B	Drugs A + B	Drugs A + B	Drugs A + B	Drugs A + B	Drugs A + B	Drugs A + B	Drugs A + B	Drugs A + B	Drugs A + B		D
E	Drugs A + C	Drugs A + C	Drugs A + C	Drugs A + C	Drugs A + C	Drugs A + C	Drugs A + C	Drugs A + C	Drugs A + C	Drugs A + C	Drugs A + C		E
F	Drugs B + C	Drugs B + C	Drugs B + C	Drugs B + C	Drugs B + C	Drugs B + C	Drugs B + C	Drugs B + C	Drugs B + C	Drugs B + C	Drugs B + C		F
G	Drugs A + B + C	Drugs A + B + C	Drugs A + B + C	Drugs A + B + C	Drugs A + B + C	Drugs A + B + C	Drugs A + B + C	Drugs A + B + C	Drugs A + B + C	Drugs A + B + C	Drugs A + B + C		G
H	Negative control (culture medium only)											UN	H

	1	2	3	4	5	6	7	8	9	10	11	12	

(i) The tested concentrations of the antibiotic were as follows: ciprofloxacin, 16–0.015 *μ*g/mL; amikacin, 32–0.031 *μ*g/mL; ceftazidime, 32–0.031 *μ*g/mL; piperacillin, 128–0.125 *μ*g/mL; imipenem, 32–0.031.

(ii) Higher dilutions of ciprofloxacin were tested in second set of experiments in double and triple combinations with other antibiotics when needed.

(iii) UN: unused well.

**Table 2:** Susceptibility of *Pseudomonas aeruginosa* clinical isolates to the antibiotics.

Isolate number	Antibiotics (*μ*g/mL)									
	CIP		AMK		CAZ		PIP		IMP	
	MIC	MBC	MIC	MBC	MIC	MBC	MIC	MBC	MIC	MBC
PA2	0.125	0.125	2	8	1	4	8	8	2	>32
PA3	0.125	0.125	2	8	8	16	16	>128	>32^R^	>32
PA9	0.5	1	4	32	8	8	8	16	2	8
PA11	>16^R^	>16	8	16	>32^R^	>32	>128^R^	>128	>32^R^	>32
PA14	2^R^	8	2	16	4	32	32^R^	32	2	>32
PA15	0.125	0.125	4	16	4	16	8	32	4	>32
PA18	0.031	0.06	2	8	2	2	8	128	8	>32
PA19	0.06	0.125	2	2	2	4	8	32	4	>32
PA20	>16^R^	>16	4	16	>32^R^	>32	>128^R^	>128	>32^R^	>32
PA21	0.125	0.25	2	8	4	4	8	32	8	>32

(i) The MIC is the minimum inhibitory concentration; MBC is defined as the concentration required to kill 99.9% of the bacteria.

(ii) The MICs of the tested antibiotics were determined by broth microdilution method.

(iii) The R letter denotes resistance of the isolate to the antibiotic based on the EUCAST MIC breakpoint guideline [21].

(iv) CIP, ciprofloxacin; AMK, amikacin; CAZ, ceftazidime; PIP, piperacillin; IMP, imipenem.

**(a):** 

Isolate number	Antibiotics (*µ*g/mL)																			
	CIP + AMK				CIP + CAZ				CIP + PIP				CIP + IMP				IMP + PIP			
	MIC	MBC	IC	IT	MIC	MBC	IC	IT	MIC	MBC	IC	IT	MIC	MBC	IC	IT	MIC	MBC	IC	IT
PA2	0.062	0.125	1	I	0.062	0.125	1	I	0.125	0.125	0	I	0.125	0.125	0	I	2	2	0	I
PA3	0.062	0.125	1	I	0.062	0.125	1	I	0.031	0.031	2	S	0.125	0.125	0	I	16	>128	0	I
PA9	0.25	1	1	I	0.25	1	1	I	0.125	0.25	2	S	0.5	1	0	I	2	8	0	I
PA11	4	16	1	I	8	>16	2	S	>16	>16	0	I	>16	>16	0	I	>32	>32	0	I
PA14	1	8	1	I	1	8	1	I	2	8	0	I	2	8	0	I	2	>32	0	I
PA15	0.125	0.125	0	I	0.062	0.125	1	I	0.062	0.125	1	I	0.062	0.125	1	I	8	>32	−1	I
PA18	0.031	0.062	0	I	0.031	0.062	0	I	0.031	0.062	0	I	0.015	0.062	1	I	32	>32	−2	A
PA19	0.031	0.125	1	I	0.062	0.125	0	I	0.062	0.125	0	I	0.031	0.125	1	I	8	>32	−1	I
PA20	2	16	1	I	>16	>16	0	I	>16	>16	0	I	16	>16	1	I	>32	>32	0	I
PA21	0.125	0.25	0	I	0.125	0.25	0	I	0.062	0.25	1	I	0.062	0.25	1	I	64	>128	−3	A

**(b):** 

Isolate number	Antibiotics (*µ*g/mL)																			
	AMK + CAZ				AMK + IMP				AMK + PIP				CAZ + IMP				CAZ + PIP			
	MIC	MBC	IC	IT	MIC	MBC	IC	IT	MIC	MBC	IC	IT	MIC	MBC	IC	IT	MIC	MBC	IC	IT
PA2	0.50	4	1	I	1	8	1	I	2	8	1	I	2	8	−1	I	0.25	4	2	S
PA3	2	8	0	I	2	8	0	I	1	8	1	I	32	>32	−4	A	2	16	2	S
PA9	1	4	2	S	1	8	1	I	2	32	1	I	2	8	0	I	4	8	1	I
PA11	4	16	1	I	8	16	0	I	4	16	1	I	>32	>32	0	I	32	>32	1	I
PA14	1	16	1	I	2	16	0	I	1	16	1	I	2	>32	0	I	0.5	4	3	S
PA15	1	4	2	S	4	16	1	I	1	4	2	S	2	32	1	I	4	16	0	I
PA18	0.50	2	2	S	1	8	1	I	2	8	1	I	8	8	−2	A	0.5	0.5	2	S
PA19	2	2	0	I	1	2	1	I	1	2	1	I	2	4	0	I	1	4	1	I
PA20	2	8	1	I	2	16	1	I	2	16	1	I	>32	>32	0	I	>32	>32	0	I
PA21	0.25	2	3	S	2	8	1	I	0.50	2	2	S	16	16	−2	A	2	4	1	I

(i) The IC (Interaction Code) is the change in the MIC of the most potent antibiotic in double or triple combination compared with the antibiotic alone.

(ii) The IC is assigned zero value if the MIC is not changed in combination, while a value of 1 or −1 is given if the MIC value decreases or increases by one dilution, respectively, in combination.

(iii) The IT (interaction type) is indifferent (I) when the IC value lies between −1 and <2, synergistic (S) if the IC value is ≥2, and antagonistic (A) if the IC value is ≤−2.

**(a):** 

Isolate number	Antibiotics (*µ*g/mL)																			
	CIP + AMK + CAZ				CIP + AMK + PIP				CIP + AMK + IMP				CIP + CAZ + PIP				CIP + CAZ + IMP			
	MIC	MBC	IC	IT	MIC	MBC	IC	IT	MIC	MBC	IC	IT	MIC	MBC	IC	IT	MIC	MBC	IC	IT
PA2	0.031	0.031	2	S	0.062	0.125	1	I	0.031	0.031	2	S	0.031	0.125	2	S	0.062	0.125	1	I
PA3	0.031	0.031	2	S	0.015	0.031	3	S	0.125	0.125	1	I	0.031	0.031	2	I^1^	0.062	0.125	1	I
PA9	0.125	0.25	3	S	0.062	0.25	3	S	0.125	0.25	2	S	0.125	0.25	2	I^1^	0.25	1	1	I
PA11	2	4	2	S	2	16	2	S	8	16	1	I	8	16	1	I	8	>16	2	I^1^
PA14	0.062	1	5	S	0.50	8	2	S	0.50	8	2	S	0.50	4	3	I^1^	1	8	1	I
PA15	0.031	0.031	2	S	0.015	0.125	3	S	0.125	0.125	0	I	0.125	0.125	0	I	0.015	0.062	3	S
PA18	0.0078	0.062	2	S	0.0078	0.062	1	I	0.031	0.062	0	I	0.007	0.015	2	S	0.015	0.062	1	I
PA19	0.015	0.031	2	S	0.015	0.031	2	S	0.062	0.125	0	I	0.015	0.031	3	S	0.031	0.125	1	I
PA20	1	8	2	S	2	16	1	I	1	16	2	S	>16	>16	0	I	16	>16	1	I
PA21	0.015	0.062	3	S	0.031	0.25	2	S	0.125	0.25	0	I	0.031	0.062	2	S	0.062	0.25	1	I

**(b):** 

Isolate number	Antibiotics (*µ*g/mL)															
	CIP + PIP + IMP				AMK + CAZ + PIP				AMK + CAZ + IMP				CAZ + PIP + IMP			
	MIC	MBC	IC	IT	MIC	MBC	IC	IT	MIC	MBC	IC	IT	MIC	MBC	IC	IT
PA2	0.062	0.125	1	I	0.125	1	3	S	0.5	4	1	I	0.125	4	3	S
PA3	0.031	0.031	2	I^1^	0.5	4	2	S	2	8	0	I	2	8	2	I^1^
PA9	0.125	0.25	2	I^1^	0.50	1	3	S	0.5	4	3	S	2	8	1	I
PA11	>16	>16	0	I	4	16	1	I	4	16	1	I	32	32	1	I
PA14	2	8	0	I	0.50	8	2	S	1	16	1	I	0.25	4	4	S
PA15	0.062	0.125	1	I	0.50	4	3	S	0.5	4	3	S	2	16	1	I
PA18	0.007	0.015	3	S	0.25	1	3	S	0.25	1	3	S	0.50	0.50	2	I^1^
PA19	0.031	0.125	1	I	0.5	2	2	S	0.50	2	2	S	1	2	1	I
PA20	16	>16	1	I	2	8	1	I	2	8	1	I	32	32	1	I
PA21	0.125	0.25	0	I	0.125	1	4	S	0.25	1	3	I^1^	2	4	1	I

(i) The IC (Interaction Code) is the change in the MIC of the most potent antibiotic in double or triple combination compared with the antibiotic alone.

(ii) The IC is assigned zero value if the MIC is not changed in combination, while a value of 1 or −1 is given if the MIC value decreases or increases by one dilution, respectively, in combination.

(iii) The IT (interaction type) is indifferent (I) when the IC value lies between −1 and <2, synergistic (S) if the IC value is ≥2, and antagonistic (A) if the IC value is ≤−2.

^1^The interaction is indifferent because the IC of the triple combination is not better than the ones of the double combinations with respect to the most potent antibiotic.

**Table 5:** Summary of the outcome of double and triple combinations of the antibiotics.

Antibiotic combination	Number/type of interactions		
	Synergism	Antagonism	Indifference
CIP + AMK	0	0	10
CIP + CAZ	1	0	9
CIP + PIP	2	0	8
CIP + IMP	0	0	10
IMP + PIP	0	2	8
AMK + CAZ	4	0	6
AMK + IMP	0	0	10
AMK + PIP	2	0	8
CAZ + IMP	0	3	7
CAZ + PIP	4	0	6
CIP + AMK + CAZ	10	0	0
CIP + AMK + PIP	7	0	3
CIP + AMK + IMP	4	0	6
CIP + CAZ + PIP	4	0	6
CIP + CAZ + IMP	1	0	9
CIP + PIP + IMP	1	0	9
AMK + CAZ + PIP	8	0	2
AMK + CAZ + IMP	4	0	6
CAZ + PIP + IMP	2	0	8

CIP, ciprofloxacin; AMK, amikacin; CAZ, ceftazidime; PIP, piperacillin; IMP, imipenem.
